# A Nonlinear Model for Gene-Based Gene-Environment Interaction

**DOI:** 10.3390/ijms17060882

**Published:** 2016-06-04

**Authors:** Jian Sa, Xu Liu, Tao He, Guifen Liu, Yuehua Cui

**Affiliations:** 1 Division of Health Statistics, School of Public Health, Shanxi Medical University, Taiyuan 030001, China; 13834643051@163.com; 2 School of Statistics and Management, Shanghai University of Finance and Economics, Shanghai 200433, China; xuliu@stt.msu.edu; 3 Department of Mathematics, San Francisco State University, San Francisco, CA 94132, USA; hetao@sfsu.edu; 4 Department of Statistics and Probability, Michigan State University, East Lansing, MI 48824, USA

**Keywords:** nonlinear gene-environment interaction, sparse principal component analysis, varying-coefficient model

## Abstract

A vast amount of literature has confirmed the role of gene-environment (G×E) interaction in the etiology of complex human diseases. Traditional methods are predominantly focused on the analysis of interaction between a single nucleotide polymorphism (SNP) and an environmental variable. Given that genes are the functional units, it is crucial to understand how gene effects (rather than single SNP effects) are influenced by an environmental variable to affect disease risk. Motivated by the increasing awareness of the power of gene-based association analysis over single variant based approach, in this work, we proposed a sparse principle component regression (sPCR) model to understand the gene-based G×E interaction effect on complex disease. We first extracted the sparse principal components for SNPs in a gene, then the effect of each principal component was modeled by a varying-coefficient (VC) model. The model can jointly model variants in a gene in which their effects are nonlinearly influenced by an environmental variable. In addition, the varying-coefficient sPCR (VC-sPCR) model has nice interpretation property since the sparsity on the principal component loadings can tell the relative importance of the corresponding SNPs in each component. We applied our method to a human birth weight dataset in Thai population. We analyzed 12,005 genes across 22 chromosomes and found one significant interaction effect using the Bonferroni correction method and one suggestive interaction. The model performance was further evaluated through simulation studies. Our model provides a system approach to evaluate gene-based G×E interaction.

## 1. Introduction

Complex human diseases are rooted in genetics, but the risk is heavily influenced by the degree of exposure to certain environmental factors. The phenomenon in which the genetic influences on disease risk are modified by environmental factors is coined as gene-environment (G×E) interaction. In practice, weak environmental stimuli is less likely to cause DNA mutations. Instead, exposure to environmental changes could cause structural changes such as DNA methylation or histone modification, which plays a regulatory rule to moderate gene expressions and consequently leads to disease signals. Such epigenetic changes have been increasing, recognized as the epigenetic basis of G×E interaction [1]. Thus, identification of G×E interaction could shed novel insights into the phenotypic plasticity of complex disease phenotypes [2].

Methods for analyzing G×E interactions have been flourishing in literature. These methods are predominantly focused on a single variant based analysis, to name a few, such as the parametric methods in [3], semi-parametric methods in [4,5], and non-parametric methods in [6,7]. Given that genes are the functional units, understanding G×E interactions from a gene level perspective could shed novel insight into the disease etiology. Thus, it is crucial to develop novel statistical methods that can assess gene-based G×E interaction effects.

Methods for gene-based genetic association analysis have been extensively studied in the literature (e.g., [8,9]). The advantage of a gene-based analysis includes: (1) biologically meaningful and ease of interpretation given that genes are the functional units; (2) a reduced number of tests given the number of genes is much smaller than the number of Single Nucleotide Polymorphism (SNPs) in a genomewide scale; (3) a released computational burden. By assessing the joint function of multiple variants in a set, a novel insight into the disease etiology could be obtained. A method for gene-based gene–gene interaction has also been proposed (e.g., [10]). However, these gene-based association methods cannot be directly extended to a gene-based G×E interaction analysis.

Motivated by empirical studies, Ma *et al.* [7] pioneered a nonlinear G×E interaction model. For continuously measured environmental variables, one can assess the varying (or dynamic) patterns of genetic effects responsive to environmental changes. Thus, a better understanding of the genetic heterogeneity under different environmental conditions can be obtained. We have extended the model to a set-based framework to investigate how variants in a gene set mediated by one or multiple environment factors to affect a disease response [11]. The method was developed under a feature selection framework in which a penalized additive varying-coefficient model was developed to select important SNPs in a gene set. This framework could shed novel insight into the elucidation of the regulation mechanism of a genetic set (e.g., a pathway), triggered by environment factors. However, it is well known that variables estimated with non-zero coefficients in a variable selection setup may not be statistically significant. Thus, the method is limited since it does not give a *p*-value for each SNP. In addition, the method is still a single variant based analysis by modeling SNPs separately in a gene set.

It is thus the purpose of this work to propose a gene-based G×E interaction model considering potential nonlinear environmental modification effects on disease risk. We propose to first reduce the SNP dimension in a gene by a classical principal component analysis (PCA). Since SNPs in a gene are potentially correlated due to linkage disequilibrium (LD), a few PCs can capture the gene variability. To ease the interpretation of PCs, we propose to further conduct a sparse PCA analysis. Sparse PCs with nonzero loadings reflect the relative importance of the corresponding SNPs. These sparse PCs are then fitted into an additive varying-coefficient model. The nonlinear varying G×E effects are estimated via the nonparametric B-spline technique. By changing the B-spline basis functions, our method is able to separate linear and nonlinear G×E effect, based on which a hypothesis testing can be done to assess different components.

We propose rigorous testing procedures to assess the main effect of a gene as well as the interaction effect between a gene and an environmental variable. The method is applied to a genome-wide association study (GWAS) dataset on birth weight in a Thai population to identify important genes triggered by nonlinear modification effects of a mother’s glucose to affect the baby’s birth weight. Simulation studies are conducted to evaluate the performance of the method with perturbed data. Our method provides a quantitative framework to evaluate and test gene-based G×E interaction, triggered by the potential nonlinear environmental modification effect.

## 2. Results

### 2.1. Simulation

To check the performance of the proposed model, we conducted a simulation study. We generated SNP data by bootstrapping samples focusing on gene *NCOA5* (see the real data analysis section for details about this gene). There are 1126 individuals and 15 SNPs in gene *NCOA5*. By bootstrapping, we assumed the original sample is the population, then randomly sampled individuals with replacement with size $n_{B}$ each time. During bootstrap, all the SNP data and the mother’s glucose level (*U*) in each individual were drawn together as a vector. By doing so, we can maintain the LD structures among SNPs as well as the correlations between SNPs and *U*. The response *Y* was then generated from the following model:

$$Y={\hat{\beta }}_{0}\left(U\right)+\tau \sum_{k=1}^{4}{\hat{\beta }}_{k}\left(U\right){\tilde{w}}_{k}+\varepsilon ,$$

where ${\hat{\beta }}_{0}\left(u\right)$ and ${\hat{\beta }}_{k}\left(u\right)$, k=1,⋯,4, are the estimators of $\beta_{0}\left(u\right)$ and $\beta_{k}\left(u\right)$ based on the real data for gene *NCOA5*, ${\tilde{w}}_{k}$ is the *k*th sparse PC in the bootstrapped samples with size $n_{B}$, and *ε* is the error term following a normal distribution with mean 0 and variance $c{\hat{\sigma }}^{2}$, where *c* is a constant controlling the size of the variance, and ${\hat{\sigma }}^{2}$ is the estimated variance in real data based on gene *NCOA5*. *τ* is a constant to control the effect size of the model. When τ=0, we can assess the empirical false positive rate. When τ>0, we can assess the testing power and we expect the power increases as *τ* increases. We set the bootstrap sample size as $n_{B}=200,\;500,\;1000$, and the constant c=1, 2, 3 to check the finite sample performance of the proposed method. Specifically, we were interested in evaluating the false positive control and the power of detecting association under different sample sizes and error variances.

As a comparison, we also analyzed the data with the VC-PCR model (4), and a simple linear regression model with a linear G×E interaction form, *i.e.*,

(1) $$Y=\alpha_{0}+\alpha_{1}U+\sum_{k=1}^{15}\beta_{1k}G_{k}+\sum_{k=1}^{15}\beta_{2k}UG_{k}+\varepsilon ,$$

where $G=(G_{1},\cdots ,G_{15})$ are the 15 SNPs in gene *NCOA5*, and *ε* is an error following a normal distribution with mean 0 and finite variance. We conducted the overall SNP effect test by testing: $H_{L,0}^{G}:\beta_{11}=\cdots =\beta_{115}=0$ and $\beta_{21}=\cdots =\beta_{215}=0$, and the SNP×E interaction effect test by testing $H_{L,0}^{I}:\beta_{21}=\cdots =\beta_{215}=0$. Let $\alpha ={\left(\alpha_{0},\alpha_{1}\right)}^{T}$, $\beta_{1}={\left(\beta_{11},\cdots ,\beta_{1K}\right)}^{T}$, $\beta_{2}={\left(\beta_{21},\cdots ,\beta_{2K}\right)}^{T}$, K=15, and $\beta ={\left(\beta_{1}^{T},\beta_{1}^{T}\right)}^{T}$. Denote LRT for testing $H_{L,0}^{G}$ by $L_{L}^{O}=-2\left(\ell_{0}\left(\hat{\alpha }\right)-\ell_{1}\left(\hat{\alpha },\hat{\beta }\right)\right)$, and LRT for testing $H_{L,0}^{I}$ by $L_{L}^{I}=-2\left(\ell_{0}\left(\hat{\alpha },{\hat{\beta }}_{1}\right)-\ell_{1}\left(\hat{\alpha },\hat{\beta }\right)\right)$, where $\ell_{0}\left(\hat{\alpha }\right)$ is the log-likelihood under $H_{L,0}^{G}$, $\ell_{0}\left(\hat{\alpha },{\hat{\beta }}_{1}\right)$ is the log-likelihood under $H_{L,0}^{I}$, and $\ell_{1}\left(\hat{\alpha },\hat{\beta }\right)$ is the log-likelihood under the full model. The LTRs $L_{L}^{O}$ and $L_{L}^{I}$ asymptotically follow a $\chi^{2}$ distribution with 30 and 15 degrees of freedom, respectively.

**Figure 1:**
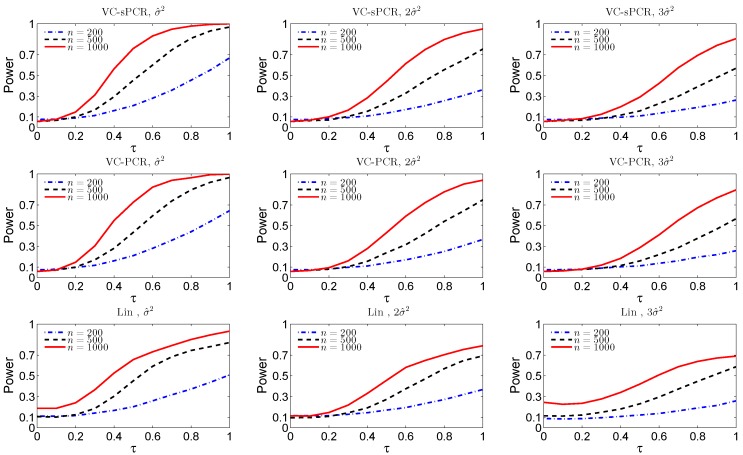
The empirical size and power functions of testing the overall genetic effect ($H_{0}^{O}$) fitted with the varying-coefficient sparse principal components regression VC-sPCR model (5) in the top row, with the VC-PCR model (3) in the middle row, and with the linear model (1) in the bottom row, under different sample sizes and error variances.

Figure 1 displays the empirical size (τ=0) and power functions (τ>0) under different sample sizes and error variances for the overall genetic effect test. The top three plots are for the overall genetic effect test fitted with the VC-sPCR model (5), the middle three plots are for the overall genetic effect test fitted with the VC-PCR model (4), and the bottom three plots are for the overall genetic effect test fitted with the linear G×E interaction model (1). As we expected, the power and size improve as the sample size increases and the error variance decreases for all the three models. For both VC-sPCR and VC-PCR models, the size and power show very similar patterns. However, since sPCR analysis assumes sparsity of the PC loadings, hence has better interpretation. As a comparison, the linear regression model has the worst performance. First of all, the size is inflated and it gets worse when sample size increases, indicating completely failing of the linear model. This is expected since the simulated interaction function is nonlinear. Moreover, the power under the linear model is also worse than the other two models. We also simulated data assuming a linear G×E interaction effect. The results show that the performance of the VC-sPCR and VC-PCR models are very similar, but their performance is slightly worse than the results by fitting a linear G×E interaction model (data not shown due to space limit). A similar phenomenon was also observed in the original nonlinear G×E interaction model [7]

**Figure 2:**
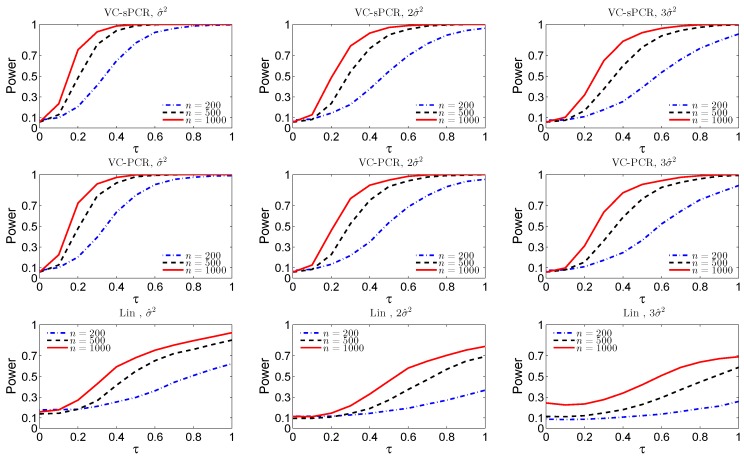
The empirical size and power functions of testing the G×E genetic effect ($H_{0}^{I}$) fitted with the VC-sPCR model (5) in the 1st row, with the VC-PCR model (3) in the 2nd row, and with the linear model (1) in the 3rd row, under different sample sizes and error variances.

For the interaction test, Figure 2 displays the empirical size and power functions under model VC-sPCR (5) the first row, under model VC-PCR (4) in the middle row, and under the linear G×E interaction model (1) in the bottom row. Again, we observed very similar patterns as for the overall genetic effect test shown in Figure 1.

### 2.2. Real Data Analysis

We applied the proposed model to a data set from the Gene Environment Association Studies initiative (GENEVA) funded by the trans-NIH (National Institute of Health) Genes, Environment, and Health Initiative (GEI), to identify important genes associated with birth weight. Fetal growth is not only determined by fetal genes but also controlled by complex interactions between fetal genes and the maternal uterine environment. In this example, we focused on the Thai population with 1126 subjects genotyped with the Omni1-Quad_v1-0_B platform (Illumina, San Diego, CA, USA) after removing potential outliers. We chose mother’s one hour OGTT (oral glucose tolerance test) glucose level (denoted as *U*) as the environmental variable in our analysis. Hypothetically, glucose from mothers can have big influence on fetal growth and such an effect can be partially captured by modeling the interaction mechanism between fetal genes and the glucose level coming from the mother.

**Figure 3:**
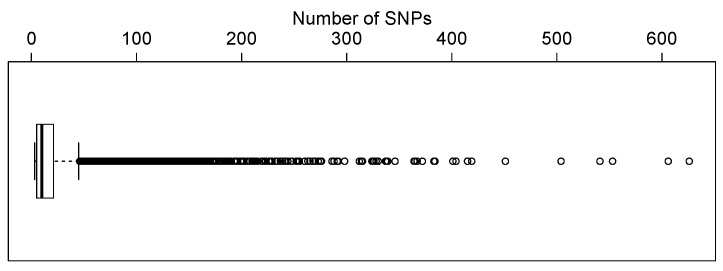
Boxplot of the number of single nucleotide polymorphisms (SNPs) in each gene.

There are total 590,913 SNPs after filtering out SNPs with minor allele frequency (MAF) <0.05, missing rate <0.05 and those deviating from Hardy–Weinberg equilibrium (*p*-value <0.001). These SNPs were then mapped to genes based on human genome builder 37 (GRCh37). We only focused on genes containing three or more SNPs in our analysis. This resulted in 12,005 genes. There are three genes containing relatively large number of SNPs (1355, 924, and 804 SNPs). Figure 3 shows the distribution of the number of SNPs in genes by excluding these three genes. The number of SNPs in most genes is less than 20.

**Figure 4:**
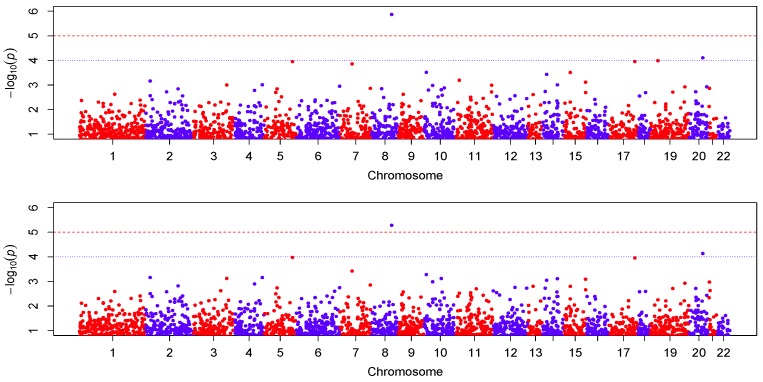
Manhattan plot of −log10(*p*-value). The **top** and **bottom** panel correspond to the result fitted with the VC-PCR and VC-sPCR model, respectively.

**Figure 5:**
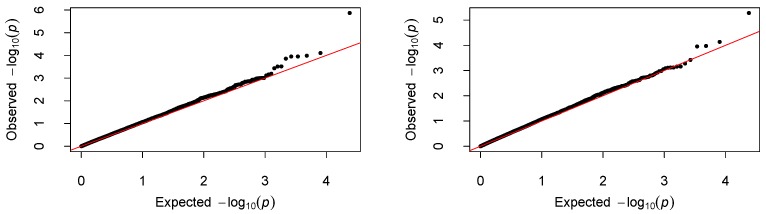
QQ-plot of the *p*-values. The **left** and **right** panel correspond to the result fitted with the VC-PCR and VC-sPCR model, respectively.

We centered the response first by subtracting the sample mean, then fitted the proposed varying-coefficient sparse PCR (VC-sPCR) model described in an earlier section to each gene, and conducted the aforementioned hypothesis testing. The number of PCs was chosen in such a way that >80% of gene variance can be explained by these PCs. As a comparison, we also fitted a regular varying-coefficient PCR (VC-PCR) model without assuming sparsity of the PC loadings. We first tested $H_{0}^{O}:\beta_{1}\left(U\right)=\cdots =\beta_{K}\left(U\right)=0$ to assess the overall genetic effect. The corresponding *p*-values are denoted by $p_{pc}^{O}$ for VC-PCR model and $p_{spc}^{O}$ for VC-sPCR model (see Table 1). Figure 4 shows the Manhattan plot of the *p*-values for the two models. The top panel is for the results analyzed with the VC-PCR model, and the bottom panel is for the results analyzed with the VC-sPCR model. The vertical axis is the −log10(*p*-value) and horizontal axis shows the genes in 22 autosome chromosomes. The two models give quite consistent signals across all the genes. If we applied a Bonferroni threshold (−log10(0.05/12005) = 5.38) at a 0.05 genome-wide significance level, only one gene (*ANGPT1*) on chromosome 8 shows significance. If we lowered the threshold to 1 × $10^{-4}$, then one gene (*NCOA5*) on chromosome 20 shows suggestive significance. The QQ-plots of the *p*-values for the two models are given in Figure 5. As we can see that no obvious departure from the diagonal line is observed, indicating no inflation of the *p*-values.

Table 1 lists the two genes along with the gene name (Gene), chromosome (Chr), the number of PCs ($N_{PCs}$), the number of SNPs ($N_{SNPs}$) within each gene, and the *p*-values of different tests. The *p*-values for testing $H_{0}^{I}$ are denoted by $p_{pc}^{I}$ for the VC-PCR model and $p_{spc}^{I}$ for the VC-sPCR model, and the *p*-values for testing $H_{o}^{M}$ are denoted by $p_{pc}^{M}$ and $p_{spc}^{M}$, respectively. The test results indicate that both the main and G×E interaction effects are significant for the two genes. For gene *NCOA5*, the G×E interaction effect is stronger (*p*-value = 1.5 × $10^{-4}$) than the main effect (*p*-value = 3.29 × $10^{-3}$).

For those sparse PCs (sPCs) in each gene, we further tested the significance of each sPC. The results show that three out of seven sPCs are significant for gene *ANGPT1*, and three out of four sPCs are significant for gene *NCOA5*. The sparse PCs along with the *p*-values, and the loadings are given in the Supplementary File. In the file, we also listed those 67 SNPs in gene *ANGPT1* and 15 SNPs in gene *NCOA5* along with the sparse loadings for each SNP.

**Figure 6:**
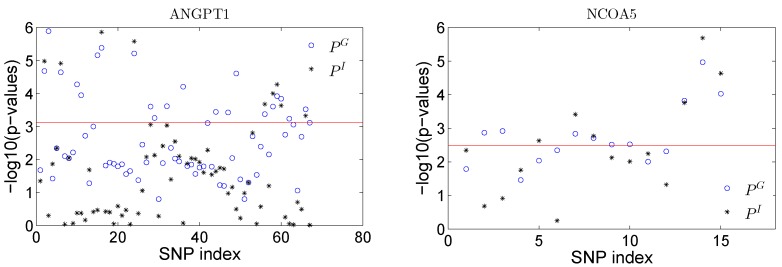
Plot of −log10(*p*-values) for testing the effect of total SNP effect ($P^{G}$) and the SNP×E interaction effect ($P^{I}$) in gene *ANGPT1* (**left panel**) and *NCOA5* (**right panel**).

As we illustrated earlier, the proposed sparse PCs can ease the interpretation given the sparse loadings of the PCs. We conducted a single SNP test by fitting the following linear model,

(2) $$Y=\alpha_{1}U+\alpha_{2}G+\alpha_{3}GU+\varepsilon ,$$

where G={0,1,2} is the SNP variable assuming an additive coding. We tested the total SNP effect by testing: $H_{0}^{G}:\alpha_{2}=\alpha_{3}=0$ and the SNP×E interaction effect by testing $H_{0}^{I}:\alpha_{3}=0$. The corresponding *p*-values using a likelihood ratio test are denoted as $p^{G}$ and $p^{I}$ and are plotted in Figure 6 for all SNPs in both genes. We can obtain some insights about the significant sPCs from the results. Take gene *NCOA5* as an example: the testing of a single sPC shows that PC1, PC2 and PC4 are significant at the 0.05 significance level. When checking the loadings for PC4, only the last three SNPs have large loadings, and the other SNPs have zero loadings on this PC. Thus, these three SNPs can be represented by PC4. A single SNP test shows that these three SNPs have the strongest effect among all the SNPs in terms of both overall and interaction effects (see Figure 6). By the sparse representation of the PCs, we have nice interpretation about the significance of these sPCs. Note that the single SNP analysis conducted here is trying to illustrate the idea of the proposed sPCR analysis and to demonstrate whether non-zero loadings make any practical sense in real analysis.

**Figure 7:**
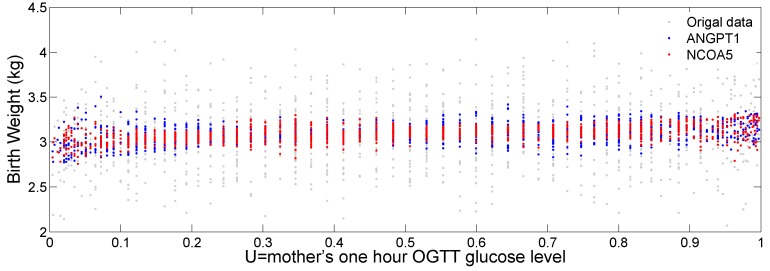
Scatter plot of the fitted birth weight against the glucose level *U*. The gray dots represent the real data. The blue and red dots represent the fitted birth weight for gene *ANGPT1* and *NCOA5*, respectively.

Figure 7 plots the original birth weight (grey dots), the fitted birth weight for gene *ANGPT1* (blue dots) and *NCOA5* (red dots) against the transformed glucose level (*U*). We can see a slightly increasing trend of fitted birth weight for the two genes as the mother’s glucose level increases. We can also see a varying pattern of the fitted values against *U*, indicating potential nonlinear interaction of the two genes with the mother’s glucose level to affect baby’s birth weight.

Gene *ANGPT1* encodes a secreted glycoprotein that belongs to the angiopoietin family and plays an important role in vascular development and angiogenesis. SNP rs2507800 in this gene has been shown to be associated with low birth weight and small-for-gestational-age infants [12]. In our analysis, this SNP did not show significant association with birth weight. This might be due to the genetic heterogeneity of birth weight for the analyzed Thai population. However, our gene-based interaction model did find evidence of association at the gene level with birth weight. Gene *NCOA5* has been shown to be associated with diabetes [13]. Many studies have shown that children born with low birth weight are associated with increased risk of developing type 2 diabetes (T2D) in adulthood [14]. Several GWAS studies have identified genetic factors associated with T2D and birth weight (e.g., [15]). As our analysis is focused on the Thai population, different from the previous GWAS report, it is possible that this gene shows significance only in the Thai population. Further studies are need to confirm this result. In addition, since we only analyzed genes containing more than two SNPs, we did not have a comprehensive coverage of all the SNP variants in our analysis. Thus, we could miss potential signals reported in other work simply because we did not analyze those variants. Our gene-based interaction analysis indicates the potential importance of these two genes on birth weight. Follow up studies will be conducted to verify the role of these two genes in other populations.

## 3. Discussion

Gene-based association analysis has been proposed to identify genes (containing multiple SNP variants) associated with complex diseases (e.g., [8,9]). Given that genes are the functional units, identifying a gene-based G×E interaction effect could shed novel insights into the genetic machinery of complex diseases. Evidenced by empirical studies and motivated by previous nonlinear G×E interaction models, in this paper, we proposed a varying coefficient model to identify gene-based gene-environment interactions, in which we allow for nonlinear influences of environmental changes on genes. We applied the sparse PCA technique to first estimate the sparse loadings of PCs and to reduce the dimension of SNP variables in a gene. Tests of association and interaction effects were then done focusing on the sparse PCs. Compared to ordinary PCR analysis, the benefit of sPCR analysis is that it can trace back to SNP variants associated with the significant PCs by checking the loading estimates. As shown in the real data analysis, we achieved nice interpretation of the sparse PCs with relative importance on the corresponding SNPs carrying nonzero loadings. This nice interpretation cannot be achieved by a regular PCR analysis without shrinking the loadings.

We applied nonparametric B-spline technique to estimate the varying coefficients of sparse PCs. By changing the spline basis functions, the model allows one to separate the main and interaction effects, thus allowing easy hypothesis testing of different genetic effects. In addition, the nonparametric technique allows one to estimate the true effect according to the data while no specific structures (such as linear) are assumed. This flexible feature is important in model fitting in real applications given that the true functional form is generally unknown.

Note that the proposed method is to capture any potential nonlinear G×E effect. As shown in [7], estimating a nonlinear function with nonparametric techniques could result in lower power compared to fitting a linear function, if the true function is linear. However, when the true interaction function is nonlinear, fitting a linear model could suffer tremendously from power loss as compared with fitting a nonlinear function. In practice, one should do a model goodness-of-fit test first, then decide which model to fit. This can be easily done by testing the linearity of the nonparametric function, *i.e.*, by testing $H_{0}:\beta \left(U\right)=\gamma_{0}+\gamma_{1}U$ using a likelihood ratio test (see [7] for details). Some genes may show linear interaction effects and some may show nonlinear interaction effects. The final results of significant genes should be a combined list from both analyses.

We applied the proposed VC-sPCR model to detect gene effects in a genome-wide scale. The computation is quite fast given the number of genes is much smaller than the number of SNPs. By focusing on genes as testing units, our model is biologically meaningful and statistically attractive. The sparse loadings of the identified PCs also enjoy nice interpretation property. Our method can be further viewed as a systems genetic approach by assessing the effect of variants in a gene as a whole. It can be easily extended to model genes in a pathway and identify pathway-environment interaction effects from a systems genetics perspective.

In the real data analysis, we identified one gene that passed the genome-wide significance level and found one suggestive gene. The results of single SNP based analysis (Figure 6) agree with the non-zero loadings of the identified sPCs. Based on our model, if a sparse PC is statistically significant, then SNPs with non-zero loadings in that sPC should be important and contribute to the effect of the sPC. Figure 6 matches the results in the supplemental file very well. The real data analysis demonstrates the utility of the proposed method. However, one has be to cautious about the statistical significance and biological significance. Further experimental validation is needed to confirm that the identified gene(s) has(have) real biological meaning.

## 4. Methods and Materials

### 4.1. The Model

Let *Y* be a complex quantitative trait. Consider a gene which contains *p* SNP variants, denoted by $G={\left(G_{1},\cdots ,G_{p}\right)}^{T}$. Let *U* denote an environmental variable that is continuously measured. We further assume that *U* (non)linearly modifies the gene effect to affect *Y*. Following [7], the relationship between *Y* and {G,U} can be modeled by the following additive varying coefficient (VC) model, *i.e.*,

(3) $$Y=\sum_{j=0}^{p}\beta_{j}\left(U\right)G_{j}+\varepsilon ,$$

where $G_{0}$ is a column of 1s. The varying coefficients $\beta_{j}\left(\cdot \right)$ are typically estimated through nonparametric techniques such as B-splines. With the spline expansion, the number of unknown parameters for each β(U) can be large depending on the number of interior knots and the spline order. To assess the gene effect, one can test $H_{0}:\beta_{1}\left(\cdot \right)=\cdots =\beta_{p}\left(\cdot \right)=0$.

Given that the number of SNP variables in a gene could be large, model (3) could easily run into the issue of “curse of dimensionality". In addition, the large number of parameters could end up with a large degree of freedom, hence reduced power to detect the interaction effect. One solution to this problem is to do a principal component analysis for SNPs in a gene to reduce the SNP dimension. Let $W_{1},\cdots ,W_{p}$ denote the principal components that are linear combinations of the original *U* variables. By selecting the first *K* PCs, which explain ≥80% of the total variance, the model (3) can be rewritten as:

(4) $$Y=\beta_{0}\left(U\right)+\sum_{k=1}^{K}\beta_{k}\left(U\right)W_{k}+\varepsilon .$$

PCA based analysis has been proposed to assess the association of an SNP set [16]. Due to linkage disequilibrium among SNPs within a gene, a PCA analysis can substantially reduce the dimension of a gene. However, the PCA based dimension reduction method faces the issue of interpretability. For example, if some PCs are significantly associated with the trait *Y*, how one can tell which SNPs contribute to the significant effect of the corresponding PCs. To aid the interpretation of the results, we propose to conduct a sparse PCA analysis first. Let ${\tilde{W}}_{1},\cdots ,{\tilde{W}}_{K}$ denote the first *K* sparse principal components, then model (4) can be rewritten as,

(5) $$Y=\beta_{0}\left(U\right)+\sum_{k=1}^{K}\beta_{k}\left(U\right){\tilde{W}}_{k}+\varepsilon .$$

Then, testing gene association modified by the environmental variable *U* can be formulated as $H_{0}:\beta_{1}\left(\cdot \right)=\cdots =\beta_{K}\left(\cdot \right)=0$ based on model (5). We refer model (4) as the varying-coefficient principal component regression (VC-PCR) model and model (5) as the varying-coefficient sparse principal component regression (VC-sPCR) model.

Methods for sparse PCA (sPCA) have been developed [17,18,19]. They all implement a penalized method to shrink the PC loadings. sPCA has been applied to genetic association studies to identify ancestry-informative markers [20]. Here, we apply the R package **elasticnet** to get the sparse PCs ${\tilde{W}}_{k},k=1,\cdots ,K$. The results of the sPCA algorithm is a list of PCs with sparse loadings. That is, unimportant SNPs will have zero loadings in the corresponding sPC, and the size of the loadings will tell the relative importance of the SNP in that sPC.

### 4.2. Parameter Estimation

We do not specify any structure for the smooth functions ${\left\{\beta_{k}\left(\cdot \right)\right\}}_{k=0}^{K}$ in model (5); rather, we estimate them with nonparametric techniques. Without loss of generality, suppose that U∈[0,1]. This can be achieved in real data by performing a data transformation for *U* if it is not uniformly distributed. Let $\delta_{k}$ be a partition of the interval [0,1], with $k_{n}$ uniform interior knots

$$\delta_{k}=\left\{0=\delta_{k,0}<\delta_{k,1}<\ldots <\delta_{k,k_{n}}<\delta_{k,k_{n}+1}=1\right\},\mathrm{for}\;k=0,\cdots ,K.$$

Let $F_{n}$ be a collection of functions on [0,1] satisfying: (1) the function is a polynomial of degree *r* or less on subintervals $I_{s}=\left[\delta_{k,s},\delta_{k,s+1}\right),s=0,\ldots ,k_{n}-1$ and $I_{k_{n}}=\left[\delta_{j,k_{n}},\delta_{j,k_{n}+1}\right)$; and (2) the functions are r−1 times continuously differentiable on [0,1]. Let $\tilde{B}{\left(\cdot \right)}_{k}={\left\{{\tilde{B}}_{kl}\left(\cdot \right)\right\}}_{l=1}^{L}$ be a set of normalized B spline basis in $F_{n}$. Then, for k=0,…,K, the VC functions can be approximated by basis functions $\beta_{k}\left(U\right)\approx \sum_{l=1}^{L}{\tilde{\lambda }}_{kl}{\tilde{B}}_{kl}\left(U\right)$, where *L* is the number of basis functions in approximating the function $\beta_{k}\left(U\right)$. With the spline expansion, model (5) becomes

(6) $$Y=\sum_{l=1}^{L}\lambda_{0l}{\tilde{B}}_{0l}\left(U\right)+\sum_{k=1}^{K}\sum_{l=1}^{L}\lambda_{kl}{\tilde{B}}_{kl}\left(U\right){\tilde{W}}_{k}+\epsilon .$$

Let $\lambda ={\left(\lambda_{0},\cdots ,\lambda_{K}\right)}^{T}$ where $\lambda_{k}={\left(\lambda_{k1},\cdots ,\lambda_{kL}\right)}^{T}$, k=0,1,⋯,K, and $\tilde{B}\left(u\right)={\left({\tilde{B}}_{1}\left(u\right),\cdots ,{\tilde{B}}_{L}\left(u\right)\right)}^{T}$. By Schumaker [21], there exists a transformation matrix Γ such that $\Gamma \tilde{B}={\left(1,{\bar{B}}^{T}\right)}^{T}$. Let $B=\Gamma \tilde{B}$. We can rewrite the coefficients to be $\beta_{k}\left(U\right)\approx \sum_{l=1}^{L}\lambda_{kl}B_{kl}\left(U\right)=\lambda_{k1}+{\bar{B}}^{T}\lambda_{k*}$, where $\lambda_{k*}={\left(\lambda_{k2*},\cdots ,\lambda_{kL*}\right)}^{T}$. By doing the transformation, the function $\beta_{k}\left(U\right)$ is partitioned into two parts, one for a constant and one for a nonlinear function. Thus, model (6) can be rewritten as:

(7) $$Y=\sum_{l=1}^{L}\lambda_{0l}{\tilde{B}}_{0l}\left(U\right)+\sum_{k=1}^{K}\lambda_{k1}{\tilde{W}}_{k}+\sum_{k=1}^{K}\sum_{l=1}^{L}\lambda_{kl*}{\bar{B}}_{kl}\left(U\right){\tilde{W}}_{k}+\epsilon .$$

Let $\lambda ={\left(\lambda_{0}^{T},\cdots ,\lambda_{K}^{T}\right)}^{T}$, where $\lambda_{k}={\left(\lambda_{k1},\lambda_{k*}^{T}\right)}^{T}$. Note that $\lambda_{k1}$ corresponds to the constant part of coefficient and $\lambda_{k*}$ corresponds to the varying part. Model (7) has nice interpretation since $\lambda_{k1},k=1,\cdots ,K$, gives the main effect of the *k*th sparse PC, and $\lambda_{k*}$ gives the corresponding (non)linear G×E interaction effect. Inference based on $\lambda_{k1}$ and $\lambda_{k*}$ can be done to assess if there is main genetic effect as well as G×E interaction effect.

Based on model (7), a least-squares technique can be applied to estimate the unknown parameters ***λ***. The B-spline coefficients ***λ*** can be estimated by

$$\begin{array}{c} \hat{\lambda }=\underset{\lambda }{arg min}R\left(\lambda \right), \end{array}$$

where $R\left(\lambda \right)=\sum_{i=1}^{n}{\left[Y_{i}-\sum_{l=1}^{L}\lambda_{0l}{\tilde{B}}_{0l}\left(U_{i}\right)-\sum_{k=1}^{K}\lambda_{k1}{\tilde{W}}_{ik}-\sum_{k=1}^{K}\sum_{l=1}^{L}\lambda_{kl*}{\bar{B}}_{kl}\left(U_{i}\right){\tilde{W}}_{ik}\right]}^{2}$. When the number of PCs is relatively large, it is computationally infeasible to select both the number of interior knots (*N*) and the order of basis function (*r*) for each PC. Therefore, we first select *N* based on the marginal function $\beta_{0}\left(u\right)$. Bayesian Information Criterion (BIC) is used to select *N* and *r* with the marginal only model $E\left[Y|X,U\right]=\beta_{0}\left(U\right)$. Specifically, we minimize the following criterion:

$$\begin{array}{c} \left(N,r\right)=\underset{N\in \{2,3,4,5\},r\in \{1,2,3\}}{arg min}\log\left(n^{-1}RSS\left({\overset{ˇ}{\lambda }}_{0}\right)\right)+n^{-1}\log\left(n\right)\left(N+r\right), \end{array}$$

where $RSS\left({\overset{ˇ}{\lambda }}_{0}\right)=\sum_{i=1}^{n}{\left\{Y_{i}-{\overset{ˇ}{\beta }}_{0}\left(U_{i}\right)\right\}}^{2}$, and ${\overset{ˇ}{\beta }}_{0}\left(u\right)$ is the estimate based on model $E\left[Y|X,U\right]=\beta_{0}\left(U\right)$. The selected knots *N* is then fixed when estimating functions $\beta_{k}\left(\cdot \right),k=1,\cdots ,K$, to save computational time. We use the similar BIC criterion to select the order of basis function when estimating each function $\beta_{k}\left(\cdot \right),k=1,\cdots ,K$.

### 4.3. Hypothesis Testing

Once the parameters are estimated, we proceed to test if there is a gene effect associated with the disease trait by testing the hypothesis $H_{0}:\beta_{1}\left(\cdot \right)=\cdots =\beta_{K}\left(\cdot \right)=0$. This is equivalent to test

(8) $$\begin{array}{c} H_{0}^{O}:\lambda_{1}=\cdots =\lambda_{K}=0\;\text{v.s.}\;H_{1}^{O}:\text{at least one is not equal zero.} \end{array}$$

We term this test as the overall gene effect test. We adopt the log-likelihood ratio test (LRT) to conduct the hypothesis testing. Under $H_{0}^{O}$, we can estimate $\lambda_{0}$ by

$$\begin{array}{c} {\hat{\lambda }}_{0}=\underset{\lambda_{0}}{arg min}R\left(\lambda_{0}\right), \end{array}$$

where $R\left(\lambda_{0}\right)=\sum_{i=1}^{n}{\left[Y_{i}-B{\left(U_{i}\right)}^{T}\lambda_{0}\right]}^{2}$. The LRT is defined as $L^{O}=-2\left(\ell_{0}\left({\hat{\lambda }}_{0}\right)-\ell_{1}\left(\hat{\lambda }\right)\right)$, where $\ell_{1}\left(\hat{\lambda }\right)$ is the log-likelihood under the full model. $L^{O}$ asymptotically follows a $\chi^{2}$-distribution with KL degrees of freedom. Failure to reject $H_{0}^{O}$ indicates that the effects on *Y* are not significant. Note that, although our main interest is to assess the significance of G×E effect, testing the overall gene effect is the first step to start with. Only when the above null hypothesis is rejected, one continues to the next step to test the significance of G×E effect, as stated in the following.

If one rejects $H_{0}^{O}$, it implies that the gene is significantly associated with the trait *Y*. To further assess if a significant G×E interaction effect exists, we propose to test the following hypothesis:

(9) $$\begin{array}{c} H_{0}^{I}:\lambda_{1*}=\cdots =\lambda_{K*}=0\;\text{v.s.}\;H_{1}^{I}:\text{at least one is not equal zero.} \end{array}$$

Again, a likelihood ratio test is applied which asymptotically follows a $\chi_{K(L-1)}^{2}$ distribution. If $H_{0}^{I}$ is rejected, then one can proceed to test which component is significant by applying the same likelihood ratio test idea. Failure to reject $H_{0}^{I}$ indicates no significant gene-based G×E interaction.

One can also test if a main gene effect on the trait *Y* exists by testing the hypothesis: $H_{0}^{M}:\lambda_{11}=\cdots =\lambda_{1K}=0$. Failure to reject the null indicates no significant main effect of the tested gene. Otherwise, one can proceed with assessing which PC has a significant main effect.

**Remark 1.**  With the sparse loadings of each PC, we have a nice interpretation of the results. For example, suppose the first PC has a significant main and interaction effect after testing $H_{0}^{M}$ and $H_{0}^{I}$. Then, we can go back to check the loadings of each SNP in that PC. Since only SNPs with non-zero loadings contribute to the PC, it implies that they are associated with the trait Y. Based on the loadings of the significant sPCs, we can make interpretation of the gene result by tracing back to individual SNPs. A regular PCR analysis will not lead to this nice interpretation in terms of individual SNP effects.

## 5. Conclusions

We proposed a gene-based nonlinear gene-environment interaction model. The model treats each gene as a unit to identify how an environmental variable nonlinearly modifies a gene effect to affect disease risk. In addition, we incorporated the sparse PCA analysis into the gene model, hence the sparse coefficient loadings imply the relative contribution of individual SNPs. With the method, one can do: (1) a gene based G×E analysis; (2) identify the relative contribution of single SNPs in each gene; and (3) detect any potential nonlinear G×E effect. Our method provides a testable framework to understand G×E interaction from a gene-centric perspective.

## Figures and Tables

**Table 1:** List of genes with *p*-value <1 × $10^{-4}$ for testing the overall genetic effect.

Gene Symbol	Chr	$n_{PC}$	$n_{SNP}$		$p_{pc}^{O}$	$p_{spc}^{O}$	$p_{pc}^{I}$	$p_{spc}^{I}$	$p_{pc}^{M}$	$p_{spc}^{M}$
*ANGPT1*	8	7	67		1.36 × $10^{-6}$	5.24 × $10^{-6}$	4.79 × $10^{-4}$	2.06 × $10^{-3}$	4.67 × $10^{-4}$	3.04 × $10^{-4}$
*NCOA5*	20	4	15		7.85 × $10^{-5}$	7.34 × $10^{-5}$	1.03 × $10^{-4}$	1.5 × $10^{-4}$	6.41 × $10^{-3}$	3.29 × $10^{-3}$

$n_{PC}$ refers to the number of PCs that explains >80% variance; $n_{SNP}$ refers to the number of SNPs in the corresponding gene.

## Supplementary Materials

Supplementary materials can be found at http://www.mdpi.com/1422-0067/17/ 6/882/s1.

## Abbreviations

BIC: Bayesian Information Criterion
G×E: Gene-environment interaction
GWAS: Genome-wide association study
OGTT: oral glucose tolerance test
LD: Linkage disequilibrium
LRT: Likelihood ratio test
MAF: Minor allele frequency
PCR: Principal components regression
SNP: Single nucleotide polymorphism
SNP×E: SNP by environment interaction
sPC: Sparse principal components
T2D: Type 2 diabetes
VC: Varying coefficient

## References

[1] Liu L., Li Y., Tollefsbol T.O. (2008). Gene-environment interactions and epigenetic basis of human diseases. Curr. Issues Mol. Biol..

[2] Feinberg A.P. (2004). Phenotypic plasticity and the epigenetics of human disease. Nature.

[3] Guo S.W. (2000). Gene-environment interaction and the mapping of complex traits: Some statistical models and their implications. Hum. Hered..

[4] Chatterjee N., Carroll R.J. (2005). Semiparametric maximum likelihood estimation exploiting gene-environment independence in case-control studies. Biometrika.

[5] Maity A., Carrol R.J., Mammen E., Chatterjee N. (2009). Testing in semiparametric models with interaction, with applications to gene-environment interactions. J. R. Stat. Soc. B.

[6] Hahn L.W., Ritchie M.D., Moore J.H. (2003). Multifactor dimensionality reduction software for detecting gene-gene and gene-environment interactions. Bioinformatics.

[7] Ma S.J., Yang L.J., Romero R., Cui Y.H. (2011). Varying coefficient model for gene-environment interaction: A non-linear look. Bioinformatics.

[8] Cui Y.H., Kang G.L., Sun K.L., Qian M., Romero R., Fu W. (2008). Gene-centric genomewide association study via entropy. Genetics.

[9] Liu J.Z., McRae A.F., Nyholt D.R., Medland S.E., Wray N.R., Brown K.M., Hayward N.K., Montgomery G.W., Visscher P.M., Martin N.G. (2010). A versatile gene-based test for genome-wide association studies. Am. J. Hum. Genet..

[10] Li S.Y., Cui Y.H. (2012). Gene-centric gene-gene interaction: A model-based kernel machine method. Ann. Appl. Stat..

[11] Wu C., Zhong P.-S., Cui Y.H. (2016). Variable selection in varying-coefficient models for gene-environment interactions.

[12] Andraweera P.H., Dekker G.A., Thompson S.D., North R.A., McCowan L.M., Roberts C.T., SCOPE Consortium (2012). A functional variant in ANGPT1 and the risk of pregnancies with hypertensive disorders and small-for-gestational-age infants. Mol. Hum. Reprod..

[13] Liu C.Y., Feng G.S. (2014). NCOA5, a molecular link between type 2 diabetes and liver cancer. Hepatobiliary Surg. Nutr..

[14] Johansson S., Iliadou A., Bergvall N., dé Fairé U., Kramer M.S., Pawitan Y., Pedersen N.L., Norman M., Lichtenstein P., Cnattingius S. (2008). The association between low birth weight and type 2 diabetes: Contribution of genetic factors. Epidemiology.

[15] Horikoshi M., Yaghootkar H., Mook-Kanamori D.O., Sovio U., Taal H.R., Hennig B.J., Bradfield J.P., Pourcain B.S., Evans D.M., Charoen P. (2013). New loci associated with birth weight identify genetic links between intrauterine growth and adult height and metabolism. Nat. Genet..

[16] Wang K., Abbott D. (2008). A principal components regression approach to multilocus genetic association studies. Genet. Epidemiol..

[17] Zou H., Hastie T.J., Tibshirani R.J. (2006). Sparse principal component analysis. J. Comput. Graph. Stat..

[18] Witten D.J., Tibshirani R., Hastie T. (2009). A penalized matrix decomposition, with application to sparse principal components and canonical correlation analysis. Biostatistics.

[19] Shen H., Huang J.Z. (2008). Sparse principal component analysis via regularized low rank matrix approximation. J. Multivar. Anal..

[20] Lee S., Epstein M.P., Duncan R., Lin X. (2012). Sparse principal component analysis for identifying ancestry-informative markers in genome-wide association studies. Genet. Epidemiol..

[21] Schumaker L.L. (1981). Spline Functions: Basic Theory.

